# Supplementary material for: AAV-DJ is superior to AAV9 for targeting brain and spinal cord, and de-targeting liver across multiple delivery routes in mice
Source: J Transl Med. 2024 Sep 5;22:824. doi: 10.1186/s12967-024-05599-5 (PMC11375878; doi:10.1186/s12967-024-05599-5)
Supplement: Supplementary file 1 — Supplementary Material 1 [file 12967_2024_5599_MOESM1_ESM.docx]

**Supplementary Figures**


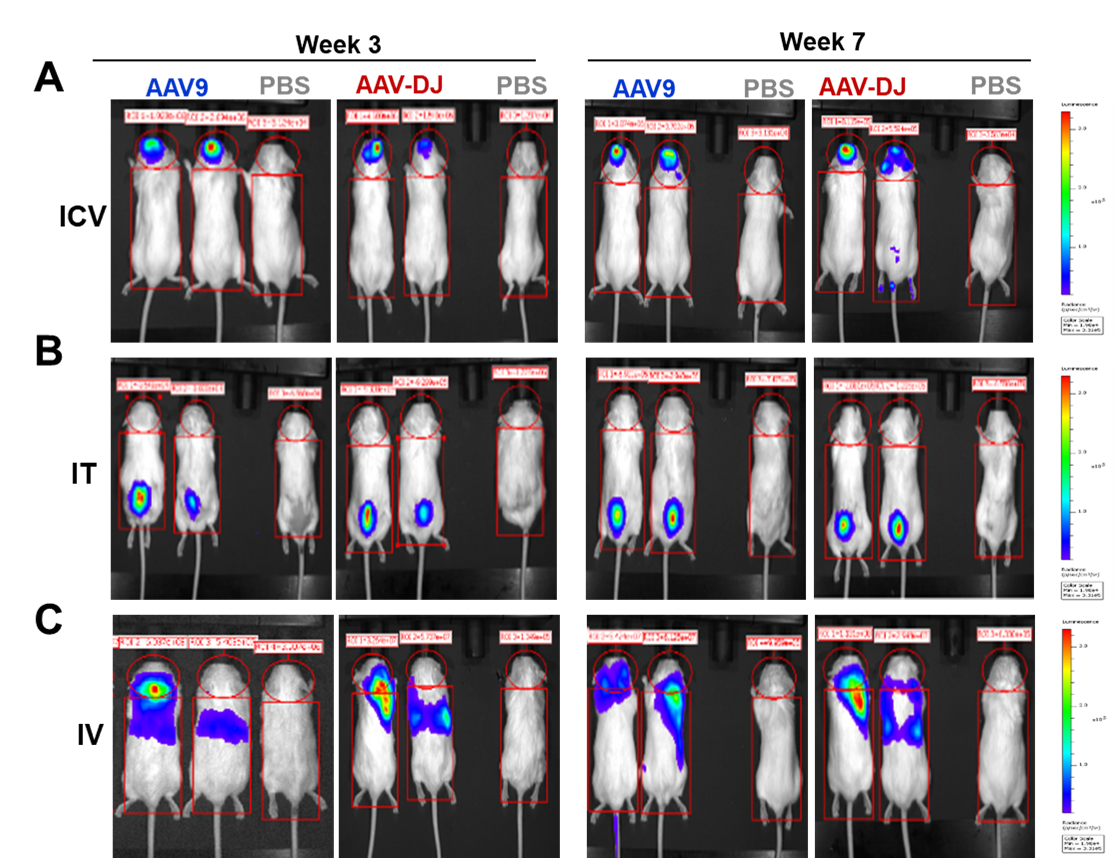


**Supplementary Figure 1:** Representative images of two mice from AAV9 and AAV-DJ groups representing each delivery route cohort for week 3 and week 7 (Figure 1). The mouse on the right-most side of every image is a no virus control.


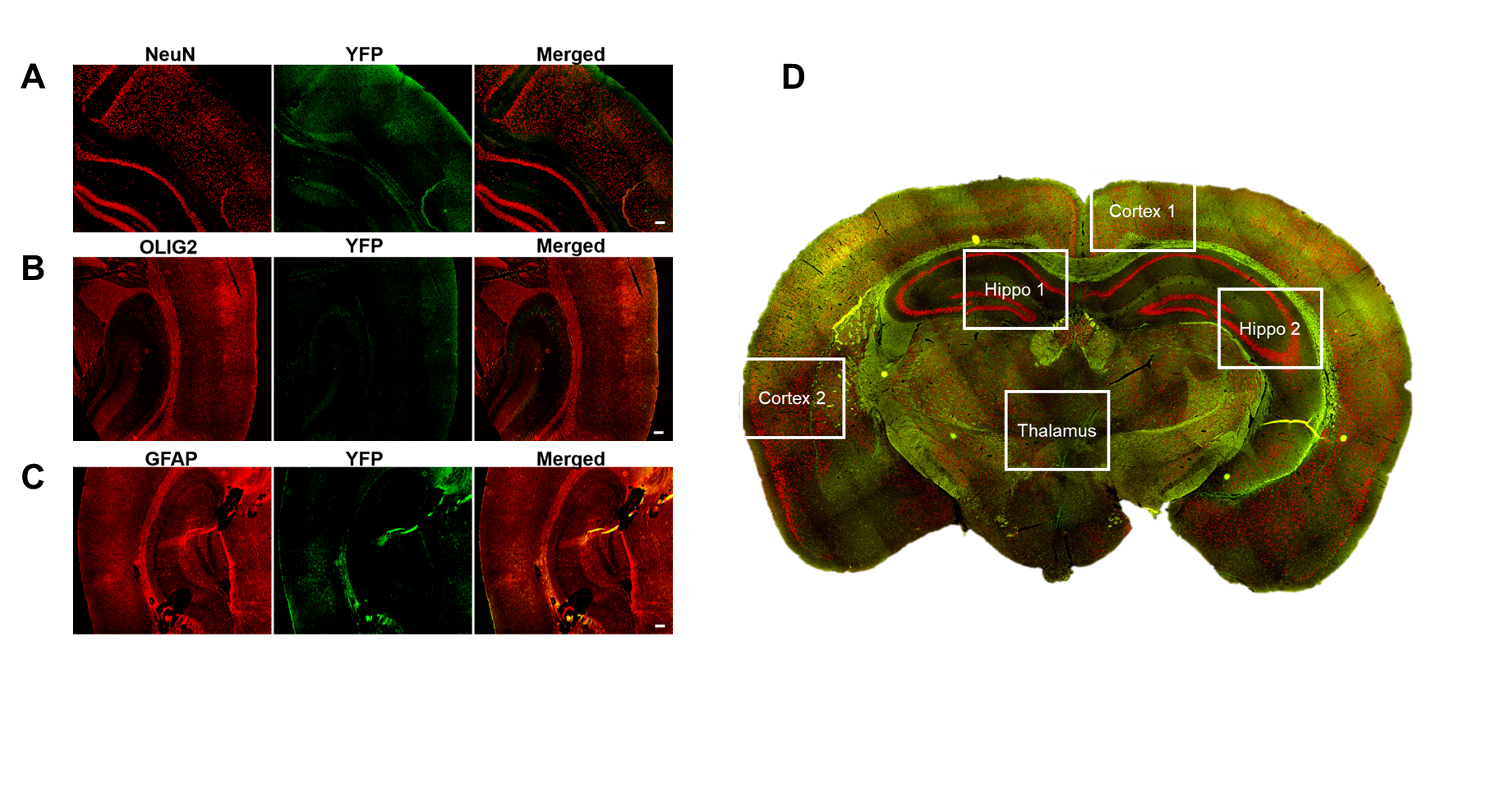


**Supplementary Figure 2:** Representative immunofluorescence image of 1xPBS injected mouse brain YFP (green) immunostaining with different markers. A) NeuN, B) OLIG2, and C) GFAP Scale bars = 100 µm. These images are negative/background controls acquired using the same exposure times and settings as those images from treated mice. D) Diagrammatic representation of the field of view selections used to obtain non-overlapping images for double positive cell quantifications from each mouse brain slice. Two images were acquired from the cortex region, two from the hippocampus, and one from the thalamus as represented on the brain section.


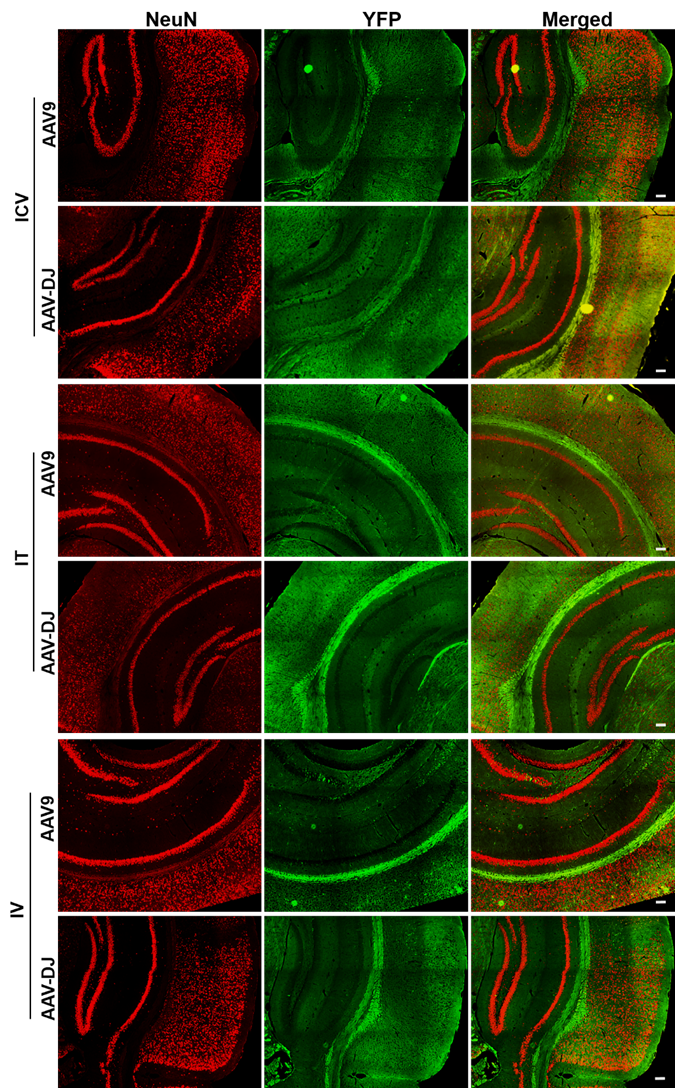


**Supplementary Figure 3:** Representative enlarged immunofluorescence images of mouse brains showing NeuN (red), YFP (green), and merged (Figure 6). Scale bars = 100 µm.


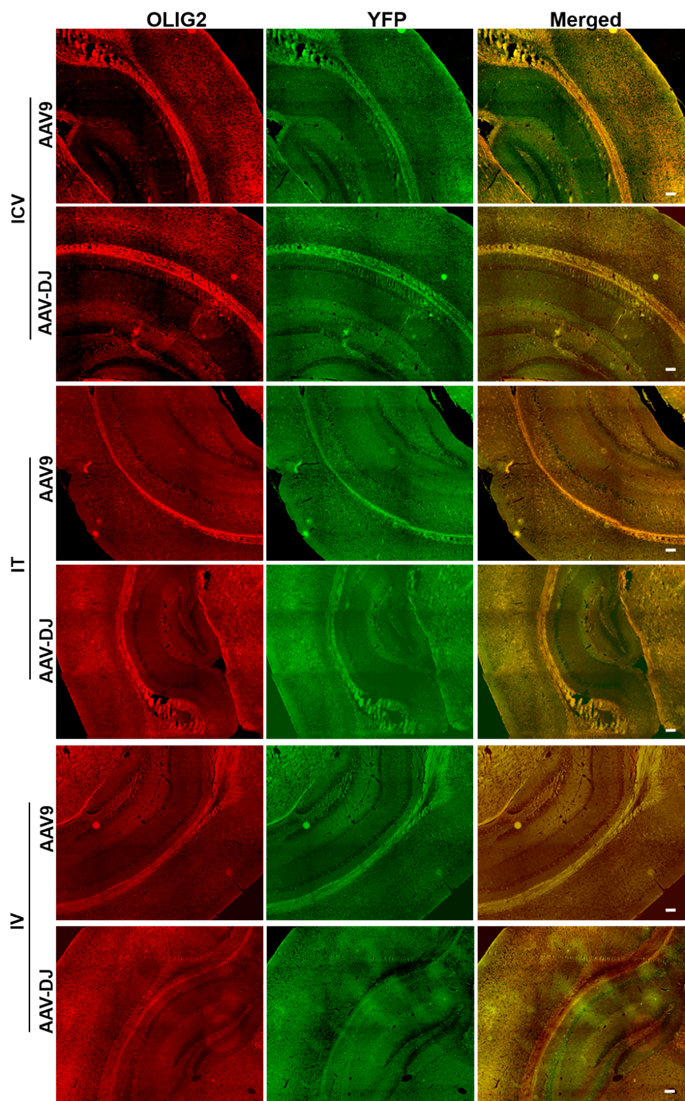


**Supplementary Figure 4:**Representative enlarged immunofluorescence images of mouse brains showing OLIG2 (red), YFP (green), and merged (Figure 7). Scale bars = 100 µm.

**
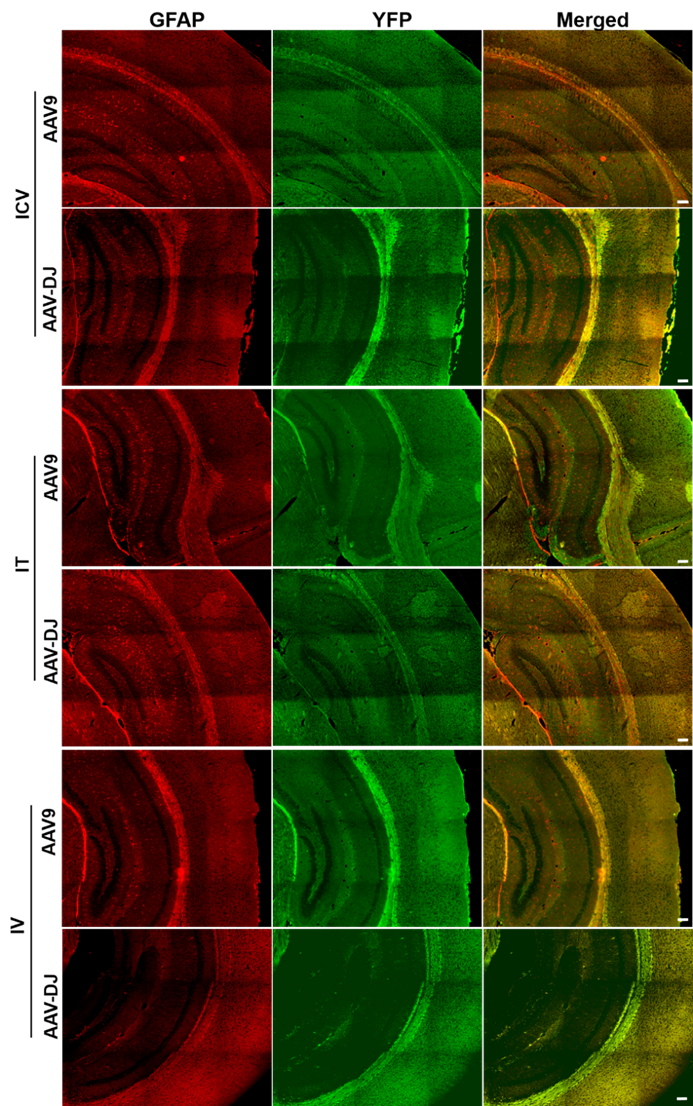
**

**Supplementary Figure 5:**Representative enlarged immunofluorescence images of mouse brains showing GFAP (red), YFP (green), and merged (Figure 8). Scale bars = 100 µm.

**
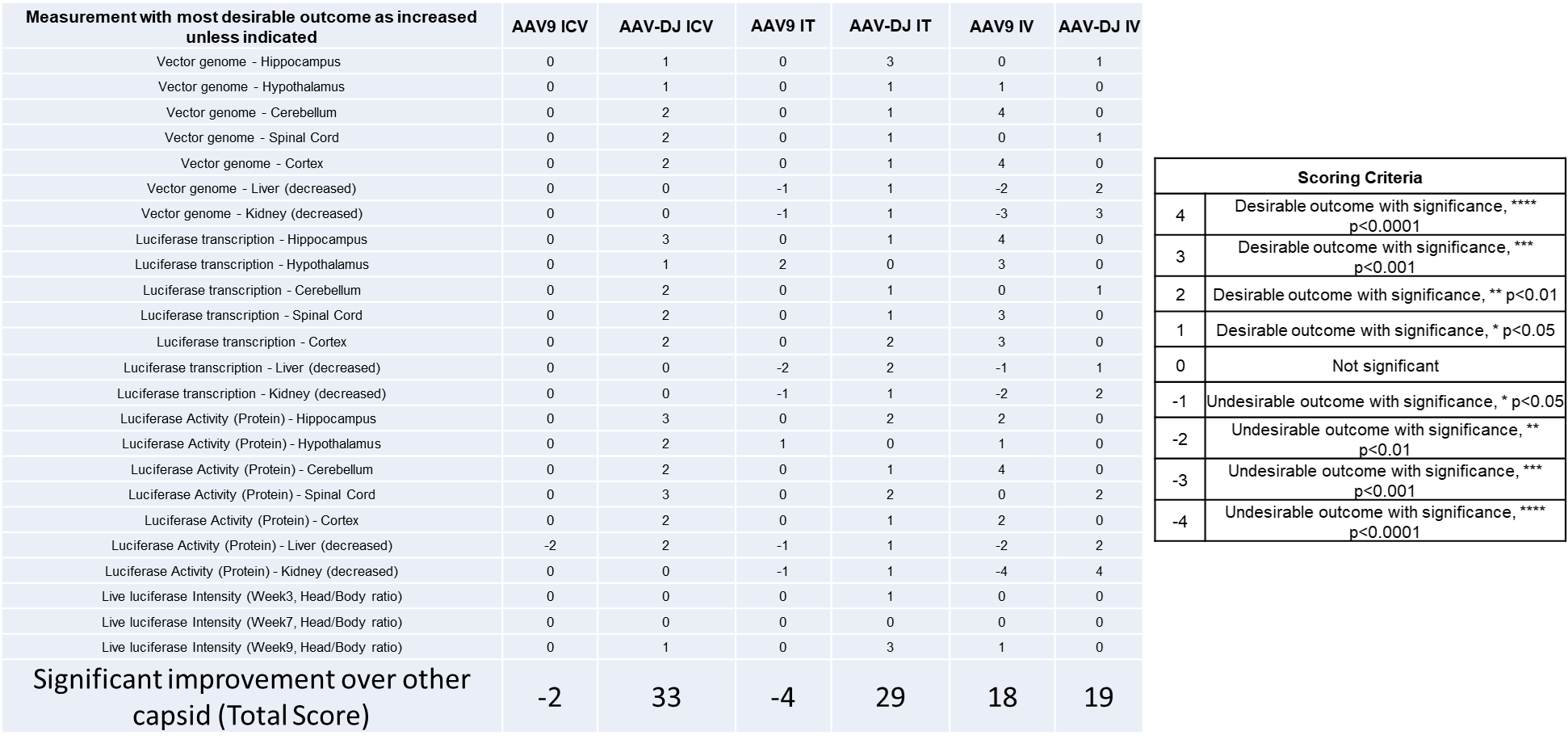
**

**Supplementary Figure 6:** AAV-mediated gene therapy capsid and delivery route comparison summary score table. Scoring criteria is indicated in the right panel.
